# Event-Related Potentials of Bottom-Up and Top-Down Processing of Emotional Faces

**DOI:** 10.15412/J.BCN.03080104

**Published:** 2017-01

**Authors:** Afsane Moradi, Seyed Abolghasem Mehrinejad, Mohammad Ghadiri, Farzin Rezaei

**Affiliations:** 1. Department of Psychology, School of Education of Psychology, University of Alzahra, Tehran, Iran.; 2. Department of Psychiatry, School of Medicine, Iran University of Medical Sciences, Tehran, Iran.; 3. Department of Psychiatry, School of Medicine, Kurdistan University of Medical Sciences, Sanandaj, Iran.

**Keywords:** Top-down processing, Bottom-up processing, Emotional faces, Event-related potential, P100, Late positive potential

## Abstract

**Introduction:**

Emotional stimulus is processed automatically in a bottom-up way or can be processed voluntarily in a top-down way. Imaging studies have indicated that bottom-up and top-down processing are mediated through different neural systems. However, temporal differentiation of top-down versus bottom-up processing of facial emotional expressions has remained to be clarified. The present study aimed to explore the time course of these processes as indexed by the emotion-specific P100 and late positive potential (LPP) event-related potential (ERP) components in a group of healthy women.

**Methods:**

Fourteen female students of Alzahra University, Tehran, Iran aged 18–30 years, voluntarily participated in the study. The subjects completed 2 overt and covert emotional tasks during ERP acquisition.

**Results:**

The results indicated that fearful expressions significantly produced greater P100 amplitude compared to other expressions. Moreover, the P100 findings showed an interaction between emotion and processing conditions. Further analysis indicated that within the overt condition, fearful expressions elicited more P100 amplitude compared to other emotional expressions. Also, overt conditions created significantly more LPP latencies and amplitudes compared to covert conditions.

**Conclusion:**

Based on the results, early perceptual processing of fearful face expressions is enhanced in top-down way compared to bottom-up way. It also suggests that P100 may reflect an attentional bias toward fearful emotions. However, no such differentiation was observed within later processing stages of face expressions, as indexed by the ERP LPP component, in a top-down versus bottom-up way. Overall, this study provides a basis for further exploring of bottom-up and top-down processes underlying emotion and may be typically helpful for investigating the temporal characteristics associated with impaired emotional processing in psychiatric disorders.

## 1. Introduction

Emotion processing includes detection and appraisal of prominent stimuli as well as regulation of emotional responses to these stimuli (Phillips, Drevets, Rauch, & Lane, 2003). Studies indicate that emotional events, particularly threatening ones, can be automatically encoded and processed (Ohman, 2005; Holmes, Nielsen, Tipper, & Green, 2009; Carlson & Reinke, 2008; Keil & Ihssen, 2004).

Studies demonstrated the process of emotional stimuli under conditions where the emotional stimuli were task-irrelevant (Eimer, Holmes, & McGlone, 2003), unattended (Vuilleumier, Armony, Driver, & Dolan, 2001; Vuilleumier, Richardson, Armony, Driver, & Dolan, 2004), or independent of conscious awareness (Whalen et al., 1998). Such stimulus-driven bottom-up processes, as evidenced by amygdala, represent an unconscious and automatic level to detect emotional cues (Anderson et al., 2003; Spezio, Adolphs, Hurley, & Piven, 2007; Whalen et al., 2004). Regarding the potential importance of emotion information to one’s safety, the bottom-up processing of emotional cues is considered to provide adaptive benefits.

Considerable research has indicated the powerful nature of emotional stimuli in automatically capturing processing resources in a bottom-up way. However, emotional stimulus could be processed consciously and voluntarily in a top-down manner (Ochsner et al., 2009; Otto, Misra, Prasad, & McRae, 2014). Top-down processing not only contributes to more in-depth understanding of emotional information, but also provides the modulation of emotional responses.

Functional magnetic resonance imaging (fMRI) studies have indicated that bottom-up and top-down processes may be mediated by distinct neural systems (Wright et al., 2008). In general, bottom-up processing associates with amygdala activation (Anderson, Christoff, Panitz, De Rosa, & Gabrieli, 2003; Phelps, 2006; Ohman, 2005; Adolphs, 2008) and top-down processing with orbital and ventromedial prefrontal cortices (Ochsner et al., 2004; Taylor, Phan, Decker, & Liberzon, 2003; Arana et al., 2003; O’Doherty, 2004; Zald & Kim, 2001). The reciprocal relationship between underlying areas involved in top-down and bottom-up processing is essential to normal emotional function. Studies have indicated that dysfunction of this neural circuit plays a critical role in creating and continuation of many psychiatric disorders (Almeida, 2009; Bishop, Jenkins, & Lawrence, 2007).

Although, imaging studies provide important clues about the spatial distinctions of these processing, temporal differentiation of top-down versus bottom-up processing of emotions, especially facial emotional expressions is unclear.

In this study, we aimed to investigate the time course of top-down and bottom-up processing of facial emotional expressions in a healthy cohort. To this purpose, the current study has primarily focused on the modulation of the well-established event-related potential (ERP) component early P100 related to capturing attention by emotionally prominent stimuli as well as late positive potentials (LPP) associated with greater processing of these stimuli.

P100 is a positive ERP component that specifically occurs about 100 ms after stimulus presentation. This wave is related to perceptual information processing in extrastriate visual regions (Allison, Puce, Spencer, & McCarthy, 1999; Hillyard, Mangun, Woldorff, & Luck, 1995). Based on research, P100 amplitude is especially enhanced for attended stimuli in comparison to non-attended ones (Hillyard, Vogel, & Luck, 1998; Luck, 2005). Studies also indicated that P100 can be affected by emotional facial processing (Iteer & Tylor, 2002; Hermanen, Ehlis, Ellgring, & Fallgatter, 2005). Effects of emotional expressions on P100 have been found as a general effect of emotional versus neutral faces (Batty & Tylor, 2003; Egar, Jedynak, Iwaki, & Skrandies, 2003) or as enhanced amplitudes in the presence of specific motional faces. For example, some studies have shown that P100 amplitudes increase in reaction to fearful face expressions (Eimer & Holmes, 2002; Luo, Feng, He, Wang, & Luo, 2010; Smith, Weinberg, Moran, & Hajcak, 2013; Williams, Palmer, Liddell, Song, & Gordon, 2006; Rellecke, Sommer, & Schacht, 2012).

The late positive potential (LPP) is a sustained positive deflection which is motivated by emotional stimuli and arises from reciprocal activation of frontal and occipital-parietal regions. LPP wave is manifested approximately 300 to 400 ms after presentation of emotional stimuli (Cuthbert, Schupp, Bradley, Birbaumer, & Lang, 2000; Moratti, Saugar, & Strange, 2011). Neuroimaging studies show that LPP component is related to activities in neural networks associated with attention and perceptual processing of motivationally important stimuli (Sabatinelli, Lang, Keil, & Bradley, 2007). Increased LPP has been observed with high arousal for pleasant and unpleasant images versus neutral ones (Cuthbert et al., 2000; Hajcak, Moser, & Simons, 2006; Hajcak & Nieuwenhuis, 2006; Keil et al., 2002; Schupp et al., 2000).

In summary, the 2 ERP components may be viable indexes, which reflect different stages of top-down and bottom-up emotional processing. Thus, we attempted to explore first, what the difference is between early perceptual processing of facial emotional expressions, as indexed by the ERP P100 component, in a top-down versus bottom-up way and second, what the difference is between later elaborative processing of facial emotional expressions, as indexed by the ERP LPP component, in a top-down versus bottom-up way.

## 2. Methods

### 2.1. Participants

Seventeen healthy female students of Alzahra University voluntarily participated in the study. All subjects were right-handed and had a normal or corrected-to-normal vision as well as normal color vision. Subjects were screened for a history of psychiatric disorders and neurological problems and were excluded from further examination in the case of reported incidents. Three subjects had to be excluded from further analyses due to high artifact of EEG data. The final sample consisted of 14 subjects (mean [SD] age=25.57[2.41] y; age range 18–30 y). Alzahra University Research Ethics Board approved the study. All participants provided written informed consents to participate in the study.

### 2.2. Stimuli

#### 2.2.1. Overt condition

The overt task was predicted to bias processing of the emotional stimuli in a top-down way. Emotional face stimuli were selected from NimStim Face stimulus set (Tottenham et al., 2009). This database includes the emotional faces of different races in Europe, Asia, and Africa (with both genders). Emotional faces utilized in this study were selected from African and European races and from both genders in equal proportion.

In the pilot study, it was observed that the subjects differentiated the emotional images with a lot of error and difficulty. Therefore, through changes in the original design, 5 separate blocks were designed. Each block consisted of 3 different type of emotions and the subjects were required to reply to each emotion with one of the left, right, or middle click of the mouse according to the instruction (so the relevant responses to 3 emotions were entered to the next calculations in each block). Each emotion had 3 responses (left, right, and middle) and the balance was established in the connection. The presentation order of the blocks was counterbalanced among participants. Each block consisted of 81 trails that eventually 81 trials were processed for each stimulus (holds responses).

**Figure 1:**
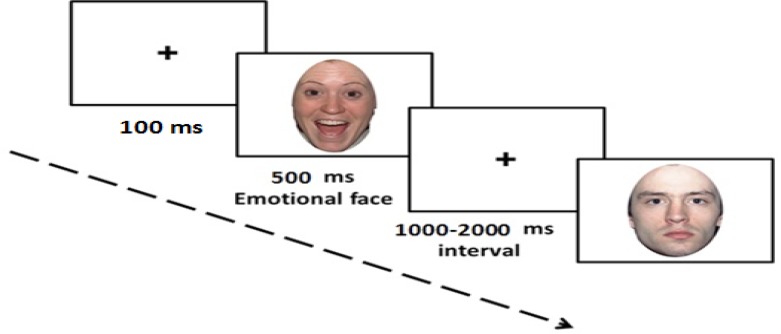
Illustration of the time course of stimulus presentation for overt condition. The time-course included a central fixation marker “+” (100 ms), visual stimulus (facial expression, 500 ms), and an inter-stimulus interval (1000–2000 ms) which during this time, participants indicated their response (affect labelling).

Each block was programmed as follows: 100 ms central fixation marker (+), 500 ms visual stimulus, and 1000–2000 ms jittered inter-stimulus interval with a central fixation marker (+), which during this time the subjects should respond. Subjects sat in front of a computer screen at a distance of 70 cm. To minimize eye blinks, subjects were requested to keep their eyes focused at the central fixation marker (+) on the computer screen. Each image was colorfully displayed with a 19×25 cm dimension. Before performing each task, participants completed practice trials to become familiar with the task and the response key configuration. Figure 1 shows the presentation of stimuli in this task.

#### 2.2.2. Covert condition

**Figure 2:**
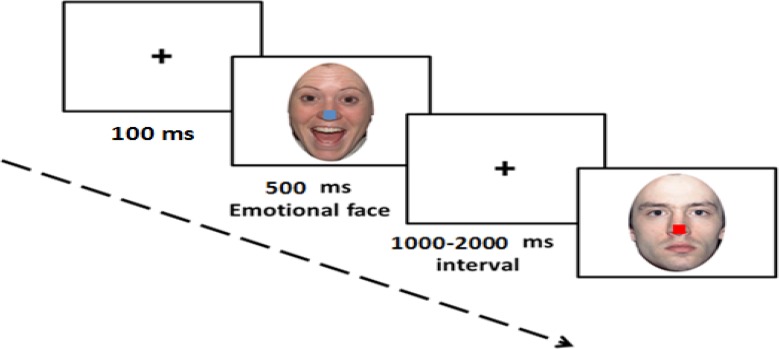
Illustration of the time course of stimulus presentation for covert condition. The time-course included a central fixation marker “+” (100 ms), visual stimulus (facial expression with colored square on nose: 500 ms), and an inter-stimulus interval (1000–2000 ms) which during this time, participants indicated their response (color discrimination).

The covert task was expected to bias processing of the emotional stimuli in a bottom-up way. In this task, the emotional face pictures, programs, and instructions were also considered like the overt task but the subjects were asked to respond to the color of squares. In this condition, small squares were placed on the nose of emotional faces (images used in the overt processing task) in 5 colors (red, yellow, blue, green, and brown) and the subjects were asked to answer the color of squares in the separate blocks (like before through clicking left, right, and middle). In this task, the place of the square was considered with a cross marker in all fixed and corresponding pictures to avoid additional eye movements. Pictures and squares were combined so that each color was placed on each 5 faces and with an equal number. Overall, 81 trials were processed for each stimulus (holds responses) in this task. The presentation order for the 2 tasks was counterbalanced among participants. Figure 2 shows how to present stimulus in this task.

### 2.3. EEG recording and analysis

Electroencephalography signals were recorded by the Mitsar system (Mitsar, Russia) using 19 active electrodes according to the international 10–20 system. EEG was sampled at 250 Hz with filtered online 0.15–50 Hz band pass and average Mastoid reference. Electrode impedance was maintained below 10 kΩ. In offline analysis, the eye blink and movements artifacts were removed through using independent component analysis. Averaged epochs included a 100 ms prestimulus baseline and a 1400 ms ERP time window. Only epochs associated with correct responses were included in averaged ERPs. Distinct ERP averages were obtained for each emotion (e.g. angry, fearful, happy, sad, and neutral) in any condition (overt and covert). The interested components in the present study included early component (P100) and LPP. P100 effect was analyzed with a time window between 80 to 120 ms over left (O1) and right (O2) electrode sites of occipital. LPP effect was analyzed with a time window between 400 and 700 ms over left (P3), middle (PZ), and right (P4) electrodes sites of parietal region.

### 2.4. Statistical analysis

Behavioral data (omission errors, commission errors, and reaction time) were analyzed through analysis of variance with repeated measures (ANOVAs), which was performed with emotions (angry, happy, fearful, sad, and neutral) and conditions (overt and covert) as repeated-measures factors.

Also, in regard to the statistical analysis of electro-physiological data, repeated measures ANOVAs were performed to analyze the amplitudes and latencies of LPP and P100. These statistical tests assumed the following arrangements: Processing condition (overt, covert)×emotion (angry, happy, fearful, sad, and neutral). We used the Bonferroni correction for any subsequent post hoc analyses.

## 3. Results

### 3.1. Behavioral results

Behavioral measures from overt and covert emotional tasks are presented in Table 1. The ANOVA with repeated measures performed on reaction time revealed a main effect for condition as (F_(1, 13)_=159.68, P<0.0001, ƞ^2^_p_=0.92), another main effect for emotion as (F_(4, 52)_=21.82, P<0.0001, ƞ^2^_p_=0.63), and an interaction effect between condition and emotion as (F_(4, 52)_=16.73, P<0.0001, ƞ^2^_p_=0.56).

**Table 1:** Behavioral measures from the overt and covert emotional tasks.

**Variables**		**Mean±SD**				

		**Angry**	**Happy**	**Fearful**	**Sad**	**Neutral**
Reaction time (ms)	Overt	726.86±97.03	734.50±117.87	881.50±92.92	825.57±86.11	757.93±112.80
	Covert	607.86±71.38	609.07±70.91	625.29±80.82	622.14±76.66	610.86±66.27
Omission (%)	Overt	2.46±1.90	2.81±2.74	2.37±1.30	2.55±1.22	1.99±1.66
	Covert	1.15±1.66	1.92±2.04	2.17±2.34	1.53±2.20	2.42±2.49
Commission (%)	Overt	0.12±0.31	0.39±0.70	0.16±0.3	0.22±0.37	0.46±0.79
	Covert	0.13±0.48	0.00±0.00	0.00±0.00	0.00±0.00	0.26±0.65

To explore the condition effect, Bonferroni-corrected t tests showed that participants were faster in the covert condition compared to the overt condition (Mean difference (MD)=170.23, P<0.0001). To explore the emotion effect, the Bonferroni-corrected t tests showed that the participants identified fearful expressions slower than angry (MD=86.04, P<0.0001), happy (MD=81.61, P<0.0001), and neutral (MD=69.00, P=0.002) expressions. Sad expressions were also associated with longer reaction times compared to angry (MD=56.07, P<0.0001), happy (MD=52.07, P=0.011), and neutral (MD=39.46, P=0.017) expressions.

Further analyses about interaction effects between emotion and condition showed that in the overt condition, the participants identified fearful expressions slower than angry (MD=154.64, P<0.0001), happy (MD=147.00, P<0.0001), neutral (MD=123.57, P=0.003), and sad (MD=55.93, P=0.027) ones. In this condition, sad expressions were also associated with longer reaction times compared to angry (MD=98.71, P<0.0001) and happy (MD=91.07, P=0.003) ones. In the covert condition, no such significant differentiation was observed between emotional face expressions.

The ANOVA with repeated measures performed on errors of omission showed no main effect for condition (F_(1, 13)_=3.02, P=0.11, ƞ^2^_p_=0.19), or main effect for emotion (F_(4, 52)_=0.48, P=0.75, ƞ^2^_p_=0.036), or interaction effect between emotion and condition (F_(4, 52)_=0.83, P=0.51, ƞ^2^_p_=0.06).

Also, the ANOVA with repeated measures performed on commission errors revealed no main effect for condition (F_(1, 13)_=4.35, P=0.06, ƞ^2^_p_=0.25), or main effect for emotion (F_(4, 52)_=1.98, P=0.11, ƞ^2^_p_=0.13), or interaction effect between emotion and condition (F_(4, 52)_=0.89, P=0.47, ƞ^2^_p_=0.06).

### 3.2. Event-related potential results

#### 3.2.1. P100 amplitude

**Figure 3:**
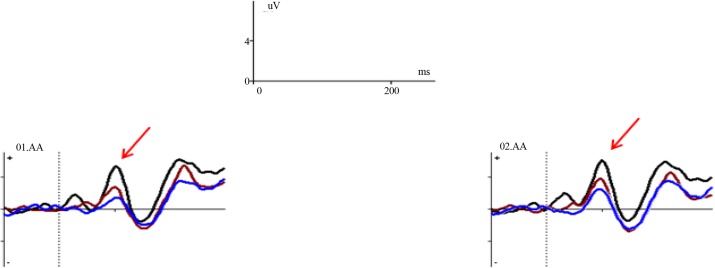
Grand-average ERPs obtained from electrodes O1 and O2 for the overt condition of happy, fearful, and angry facial expressions showing that the P100 component is the highest amplitude in the fearful (black) and happy (brown) expressions, and is the lowest in the angry expression (blue).

The descriptive indicators of the P100 amplitude in the overt and covert emotional tasks are presented in Table 2. The ANOVA with repeated measures revealed no main effect for condition (F_(1, 13)_=1.77, P=0.20, ƞ^2^_p_=0.12), but a main effect for emotion (F_(4, 52)_=5.93, P<0.0001, ƞ^2^_p_=0.31), and an interaction effect between emotion and condition (F_(4, 52)_=2.61, P=0.046, ƞ^2^_p_=0.17). To explore the emotion effect, the Bonferroni-corrected pairwise comparisons showed that fearful expressions elicited more P100 amplitude compared to anger (MD=2.23, P=0.035) and sad (MD=1.83, P=0.036) expressions. Also, further analyses with regard to interaction effects between emotion and condition showed that in the overt condition, fearful expressions elicited more P100 amplitude compared to angry (MD=3.12, P=0.004), happy (MD=2.34, P=0.005), sad (MD=2.73, P=0.005), and neutral (MD=2.89, P=0.016) expressions (Figure 3). In the covert condition, no such significant differentiation was observed between emotional face expressions.

**Table 2:** Descriptive statistics for amplitude (Hz) and latency (mv) of P100 and LPP components across overt and covert emotional tasks.

**Variables**			**Mean±SD**				

			**Angry**	**Happy**	**Fearful**	**Sad**	**Neutral**
P100	Amplitude	Overt	4.41±4.95	5.18±3.73	7.53±4.58	4.80±3.83	4.63±3.30
		Covert	3.63±4.43	4.69±4.88	4.97±4.49	4.04±5.37	4.89±5.13
	Latency	Overt	104.42±14.97	103±15.23	103.42±12.73	110.28±16.17	100.42±17.77
		Covert	97.43±13.44	98.28±12.52	101.14±14.20	98.86±11.86	100.43±11.26
LPP	Amplitude	Overt	15.18±5.40	13.97±4.66	13.56±4.63	12.71±2.85	14.97±4.98
		Covert	9.79±3.46	9.90±3.95	10.00±4.45	9.22±4.02	9.30±4.10
	Latency	Overt	533.90±70.72	535.81±70.01	550.48±95.71	516.48±78.79	531.90±73.98
		Covert	510.76±97.24	496.67±86.74	482.76±72.15	497.33±87.65	485.62±94.56

#### 3.2.2. P100 latency

The descriptive indicators of P100 latency in the overt and covert emotional tasks are presented in Table 2. The ANOVA with repeated measures revealed no main effect for condition (F_(1, 13)_=2.75, P=0.12, ƞ^2^_p_=0.17), or emotion (F_(4, 52)_=1.26, P=0.30, ƞ^2^_p_=0.09), or interaction effect between emotion and condition (F_(4, 52)_=1.83, P=0.14, ƞ^2^_p_=0.12).

#### 3.2.3. Late positive potential (LPP) amplitude

**Figure 4:**
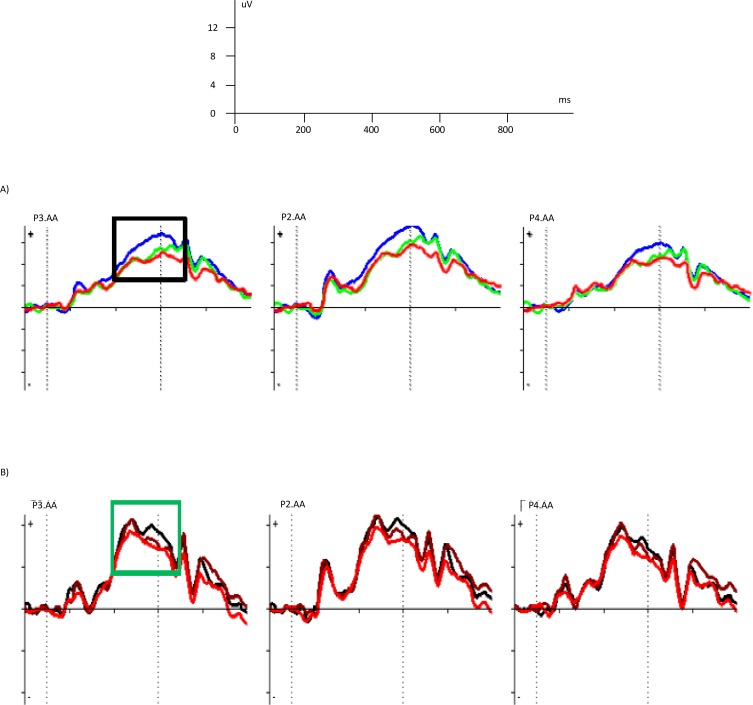
Grand-average ERPs obtained from electrodes P3, PZ, and P4 in the overt (a) and covert conditions (b). a. Grand-average LPPs in the overt condition; angry (blue), neutral (green), sad (red). It shows that the LPP component for angry expression has the highest amplitude, and for sad expression the lowest amplitude. Black square indicates the time ranges used for averaging the LPP components. b. Grand-average LPPs in the covert condition; fearful (black), happy (brown), sad (red). It shows that the LPP amplitude for the fearful and happy expressions has the highest amplitude, and for sad expression the lowest amplitude. Green square indicates the time ranges used for averaging the LPP components.

The descriptive indicators of the LPP amplitude in the overt and covert emotional tasks are presented in Table 2. The ANOVA with repeated measures revealed a main effect for condition (F_(1, 13)_=116.03, P<0.0001, ƞ^2^_p_=0.90), but no main effect for emotion (F_(4, 52)_=1.21, P=0.32, ƞ^2^_p_=0.08), and no interaction effect between emotion and condition (F_(4, 52)_=1.30, P=0.28, ƞ^2^_p_=0.09). To explore the condition effect, the Bonferroni-corrected t tests showed that the overt condition elicited more LPP amplitudes compared to covert condition (MD=4.43, P<0.0001) (Figure 4).

#### 3.2.4. Late positive potential (LPP) latency

The descriptive indicators of the LPP latency in the overt and covert emotional tasks are presented in Table 2. The ANOVA with repeated measures revealed main effect for condition (F_(1, 13)_=6.74, P<0.022, ƞ^2^_p_=0.34), but no main effect for emotion (F_(4, 52)_=0.57, P=0.69, ƞ^2^_p_=0.04), and no interaction effect between emotion and condition (F_(4, 52)_=1.62, P=0.18, ƞ^2^_p_=0.11). To explore the condition effect, the Bonferroni-corrected t tests showed that the overt condition elicited more LPP latencies compared to covert condition (MD=39.09, P=0.022) (Figure 4).

## 4. Discussion

The current study explored the time course of processing facial emotional expressions in a top-down way against bottom-up way. In this regard, we examined 2 reliable ERP indexes of emotional processing, namely P100 and LPP components, to study differences at the time course of top–down and bottom-up processing of facial emotional expressions.

Behavioral analysis of responses showed no significant difference with regard to types of errors (i.e. omission and commission) in overt and covert conditions. However, the analysis of reaction times revealed that participants were faster in the bottom-up processing compared to top-down processing. Obviously, top-down condition due to elaborative and greater cognitive processing was associated with longer reaction time. This study also found that fearful and sad faces were respectively related to the slowest reaction times spatially in the overt condition. Studies indicate that fearful faces are generally the least accurate, the latest, and the slowest ones to be identified (Calvo & Lundqvist, 2008; Palermo & Colthear, 2004).

The findings of the present research about P100 showed that the fearful expressions produced greater P100 amplitudes compared to other facial expressions. It also indicated that fearful facial expressions modulated the P100 compared to other facial expressions only within the overt condition, while no such differentiation was observed within the covert condition. This finding is consistent with previous results, as the P100 strongly influenced by fear stimulus (Luo, Feng, He, Wang, & Luo, 2010; Smith, Weinberg, Moran, & Hajcak, 2013; Williams, Palmer, Liddell, Song, & Gordon 2006; Rellecke, Sommer, & Schacht, 2012). Such a speedy response in early perceptual stage following initial stimulus detection suggests an automatically enhanced perceptual encoding of threat-related cues.

Fearful expressions probably due to the higher evolutionary relevance of threat-related stimulus, quickly capture processing resources. Therefore, attending to threatening cues such as fearful ones is evolutionarily adaptive and increases likelihood of survival. In addition, these emotion-specific modulations were only observed in the top-down processing of face expression suggesting task-driven effects. The P100 modulations are consistent with previous research results where the P100 component has been known as a characteristic of selective attention to relevant stimuli and general arousal (Luck, 2005).

Evidence has also shown that P100 amplitude is typically enhanced for attended stimuli in comparison to non-attended stimuli (Hillyard, Vogel, & Luck, 1998; Clark & Hillyard, 1996; Correa, Lupianez, Madrid, & Tudela, 2006; Handy & Khoe, 2005). Similar to mentioned research, such a finding could partly reflect the sensitivity of P100 component to attention modulations and engaging top-down mechanisms. Overall, the P100 findings showed enhanced early perceptual processing of fearful face expressions in a top-down way compared to bottom-up way.

LPP findings of the current study revealed only general effect for the task manipulation, with enhanced LPP latencies and amplitudes associated with top-down processing of emotional faces. Even the current behavior data showed slower reaction times in overt condition. The current LPP findings are consistent with previous studies indicating enhanced processing for more elaborated tasks (Van Strien, De Sonnenville, & Franken, 2010; daSilva, Crager, & Puce, 2016). It is also thought that LPP component is affected by spatial attentional deployment and task relevance (Thomas, Johnstone, & Gonsalvez, 2007). Therefore, top-down condition was associated with increased LPP latencies and amplitudes, as overt condition is task-relevant and involves greater cognitive processing.

In conclusion, early perceptual processing of fearful face expressions is enhanced in top-down way compared to bottom-up way. It also suggests that P100 may reflect an attentional bias to fearful emotions. However, no such differentiation was observed within later processing stages of face expressions, as indexed by the ERP LPP component, in a top-down versus bottom-up way. Overall, this study provides a basis for further exploring of bottom-up and top-down processes underlying emotions and may be helpful for investigating the temporal characteristics associated with impaired emotional processing in psychiatric disorders.

One of the limitations of this study was the sample which included women only. A previous research revealed gender effects in emotion processing (Hall & Matsumoto, 2004). Therefore, we suggest that future studies consider the time courses of bottom-up and top-down emotion processing among men and women to explore gender differences. Another limitation of this study was research tasks. For example, in covert task designed for bottom-up process measurement, the consciousness was not completely omitted, but involved in the processing of facial expressions without deliberate or overt attention. Since bottom-up process is an unconscious and automatic process, this covert task may not fully represent a bottom-up process. Thus, it is recommended that the tasks designed for bottom-up process measurement, be completely unconscious and stimulants be represented out of conscious awareness.
